# COMPASS: Continuous Open Mouse Phenotyping of Activity and Sleep Status

**DOI:** 10.12688/wellcomeopenres.9892.2

**Published:** 2017-04-24

**Authors:** Laurence A. Brown, Sibah Hasan, Russell G. Foster, Stuart N. Peirson

**Affiliations:** 1 Sleep and Circadian Neuroscience Institute (SCNi), Nuffield Department of Clinical Neurosciences, University of Oxford, Oxford, UK

**Keywords:** Actigraphy, mouse, circadian, sleep, PIR, EEG

## Abstract

**Background: **Disruption of rhythms in activity and rest occur in many diseases, and provide an important indicator of healthy physiology and behaviour. However, outside the field of sleep and circadian rhythm research, these rhythmic processes are rarely measured due to the requirement for specialised resources and expertise. Until recently, the primary approach to measuring activity in laboratory rodents has been based on voluntary running wheel activity. By contrast, measuring sleep requires the use of electroencephalography (EEG), which involves invasive surgical procedures and time-consuming data analysis.

**Methods**: Here we describe a simple, non-invasive system to measure home cage activity in mice based upon passive infrared (PIR) motion sensors. Careful calibration of this system will allow users to simultaneously assess sleep status in mice. The use of open-source tools and simple sensors keeps the cost and the size of data-files down, in order to increase ease of use and uptake.

**Results**: In addition to providing accurate data on circadian activity parameters, here we show that extended immobility of >40 seconds provides a reliable indicator of sleep, correlating well with EEG-defined sleep (Pearson’s r >0.95, 4 mice).

**Conclusions**: Whilst any detailed analysis of sleep patterns in mice will require EEG, behaviourally-defined sleep provides a valuable non-invasive means of simultaneously phenotyping both circadian rhythms and sleep. Whilst previous approaches have relied upon analysis of video data, here we show that simple motion sensors provide a cheap and effective alternative, enabling real-time analysis and longitudinal studies extending over weeks or even months. The data files produced are small, enabling easy deposition and sharing. We have named this system COMPASS - Continuous Open Mouse Phenotyping of Activity and Sleep Status. This simple approach is of particular value in phenotyping screens as well as providing an ideal tool to assess activity and rest cycles for non-specialists.

## Introduction

24 hour rhythms of activity and rest occur in virtually all organisms. Remarkably, these circadian (‘around a day’) rhythms persist in the absence of external stimuli, demonstrating the presence of an internal biological clock. The timing of many biological processes, including locomotor activity and sleep, are regulated by the circadian system and disruption of these rhythms has been associated with a wide range of health consequences, including cognitive impairment, metabolic and cardiovascular disease and even cancer ^1, 2^. In laboratory mice, patterns of activity and sleep provide valuable markers of health and disease. Animals display characteristic changes in physiology and behaviour associated with illness, including increased sleep, fever, weight loss and reduced social interaction ^3^, yet changes in activity and sleep are rarely used as welfare indicators ^4^. Furthermore, changes in circadian rhythms and sleep may precede symptoms in other disorders, as has been shown in neurodegenerative and neuropsychiatric disease ^5– 7^. As a result, many researchers working with laboratory mice are interested in studying longitudinal patterns of activity and sleep in disease models.

Measuring long-term locomotor activity and sleep in mice requires specialised resources and expertise, and may require surgical intervention. Cost and complexity have previously placed limits on the widespread use of such measures. Whilst many commercial systems are available, these are often targeted at those with specific research interests in circadian rhythms or sleep, and are not suitable for widespread implementation. When a potential behavioural phenotype is predicted in a transgenic model and the mice are available in another facility, the ability to examine activity and sleep patterns, non-invasively, within the home facility would be beneficial. Moving transgenic animals often requires specialist facilities for import and re-derivation of the model, a process often required to maintain health standards within facilities. Avoiding the need for such procedures would reduce the number of animals bred for research and kerb increased economic costs of the research.

A wide range of methods are used to monitor locomotor activity, such as running-wheels, beam-break or video monitoring. Whilst home cage running wheels are widely used in circadian biology ^8, 9^, it is recognised that exposure to a wheel may modify the behaviour of the animal ^10^. Beam-break methods are ideal for short duration assessment of exploratory behaviour ^11^, but require specialised caging and are therefore not suitable for long-term monitoring.

Sleep is a complex physiological process resulting in coordinated changes in locomotion, body posture and responsiveness to stimuli ^12^. Whilst sleep provides an ideal welfare indicator, it is rarely used in laboratory rodents due to the requirement of electroencephalography (EEG) and electromyography (EMG) which involve surgical intervention. Whilst the detailed study of sleep stages require EEG/EMG, a number of non-invasive correlates including video-based monitoring and piezo-electric sensors have been described which enable high-throughput assessment of sleep status, enabling total sleep, sleep timing and sleep fragmentation to be measured ^13– 15^. Whilst video monitoring has been increasingly used ^13^, such methods generate large amounts of data and are computationally intensive, making them unsuitable for large scale implementation or real-time analysis.

To address these issues, we established a minimal system for measuring activity in relation to the lighting environment. The use of microcontrollers (simple, single-chip computers) and in particular the Arduino family of open-source hardware has been discussed as a flexible solution to a number of scenarios where current lab equipment and technologies are either insufficient or prohibitively expensive ^16, 17^. From the outset, the design of the system was kept as simple as possible, whilst maintaining integration of the sensors at the level of the microcontroller. Using pyroelectric or passive infrared sensors (PIRs) provide a cheap means of measuring activity which is easily incorporated in home cages and is easily scalable.

## Methods

### Activity monitoring system

The PIR used incorporated an integrated digital amplifier (model: Panasonic EW AMN32111) alongside a light-dependent resistor (LDR, Excelitas Tech - VT90N1). The use of PIR sensitive to slight movements and with an inbuilt amplifier will help to ensure consistency of the data generated. In this regard, the essential considerations for reproducibility will be the type of sensor used, the distance from sensor to mouse and the temporal resolution of the measurements. In the system built for this paper the sensors were incorporated into simple circuits (see Supplementary material). The sensors were read using the Arduino Uno (Rev3) board, featuring an ATmega328 microcontroller ( http://arduino.cc/en/Main/ArduinoBoardUno). Printed circuit boards were designed using Fritzing ( http://fritzing.org/home/, versions up to 0.9.3b), and manufactured through their fabrication service. All other components were from Farnell ( http://uk.farnell.com/, Leeds, UK), or Mouser Electronics ( http://uk.mouser.com/, London, UK). Software (sketches) for the system was written and tested using versions 1.0.1–1.0.5 and 1.5.6–1.6.8 of the Arduino integrated development environment (IDE, available at http://arduino.cc/en/Main/Software). This software is written in Java and is both open-source and cross-platform ^18^. Data storage and visualization was carried out using Processing (also free and open-source and available for multiple platforms, see http://processing.org/). The programs detailed in the current paper work with versions up to 2.2.1.

### Positioning of sensors

The cages used in the study were either Techniplast 2154F (Techniplast S.p.A., overall size 482 × 267 × 210 mm), with a modified top and externally-mounted holders for food and water, or an MB1 mouse cage (North Kent Plastic Cages, Overall size: 450 × 280 × 130mm), with wire top. An accurate measurement of activity and rest relies on the infrared radiation from the mouse being able to reach the sensor at all times. For this reason the small gaps under the food and water hoppers on the MB1 cages were blocked using Perspex blocks and no environmental enrichment was used that could fully obscure the animal (e.g. Perspex tubes). The nesting materials used in the study were either paper-based Sizzle-Nest, or cotton fibre Nestlets (Datesand Group, Manchester, UK), with a minimum of 10g in each cage (in excess of that required for successful thermoregulation by mice with similar materials ^19^). Excessive nesting materials could potentially obscure the mouse from the sensor and nesting boxes will prevent the accurate assessment of activity and sleep. Cages require wire tops as most plastics show very low transmissivity for the wavelengths of energy detected by infrared sensors (approximately 5–10µm).

### The timing and collection of data

In order to establish an efficient system, the collection of data was based around previous work showing that periods of immobility >40s in mice were an accurate indicator of sleep ^13, 15^. Preliminary experiments with the PIRs showed that a polling time of 100msec was a good balance between capturing brief movements and overestimating movement due to the dwell-time of the PIR (the time the sensor stays active after movement stops). With 10s bins this provides 100 separate 100msec measurements and therefore a simple % activation of the sensor can be calculated for each of the 10s epochs. A serial message containing all the activity data and a single measurement of environmental light from the LDR is then sent to the PC connected via USB, where it stored alongside a timestamp. Using ISO8601 UTC (Coordinated universal time) timestamps for the data as saved to file helps to minimise future errors caused by difference in time zones and daylight savings times.

### Calculation of sleep status from activity

With data collected as a percentage activity in 10s bins, the calculation of 40s of immobility is as simple as a rolling sum over the last 4 bins. This can be achieved in many software programs, including the Microsoft Excel (using a function such as “=IF(SUM(A1:A4)=0,1,0)”). For the current study these calculations were carried out using the python PyData stack, ( http://pydata.org/), including the Pandas library (version 0.18.0) ^20^ that will allow easy import of the .csv files produced by the system, resampling and rolling-sum calculations. Examples of the processing of data are provided in a series of interactive notebooks online ( Data and software availability).

### The use of animals

All work was carried out in accordance with Animal [Scientific Procedures] Act 1986, with procedures reviewed by the clinical medicine animal care and ethical review body (AWERB), and conducted under project licence PPL 30/2812 and personal licences I459D3D59 and IDB24291F. Young-adult male wild-type C57BL/6J mice (RRID:IMSR_JAX:000664), were obtained from Envigo (Alconbury UK), with all experiments carried out when the mice were between 12 and 24 weeks of age. Animals were housed in specific pathogen free conditions, with the only reported positives on health screening over the entire time course of these studies being for *Helicobacter hepaticus* and *Entamoeba spp.* All animals were singly-housed, provided with food and water ad-libitum and maintained on a 12h light:12h dark cycle (150–200 lux, measured at the cage floor), in light-tight environmental enclosures (in groups of 6 cages). Where constant light was used to establish circadian parameters, the same intensity of 150–200 lux was used. Comparison of EEG and PIR-derived sleep was carried out within individual mice, and no mice were excluded from any group in this work.

### Comparison of EEG and PIR-estimated sleep

A telemetric transmitter (volume, 1.9cm ^3^; total weight, 3.9g; TL11M2-F20-EET; DSI, St. Paul, MN, USA) connected to electrodes for continuous EEG and EMG recordings was implanted in 4 adult male C57BL/6J mice (15.8 ± 0.6 weeks old, mean ± S.E.M.) as described in (and adapted from Hasan *et al*., 2014) ^21^. Briefly, in mice under anesthesia (isoflurane induction 4.5%, maintenance 0.7–2.25%), 2 stainless-steel EEG electrodes (length of screw shaft, 2.4mm; outer diameter of screw thread, 1.19mm) were implanted epidurally over the right frontal and parietal cortices ^22^ and connected to the telemetry transmitter via medical grade stainless-steel wires (surrounded by silicone tubing). The EEG electrodes and connections to the subcutaneous wiring were covered with dental cement (RelyX Arc; Kent Express, Kent, UK). Two EMG stainless-steel leads were inserted into the neck muscle ~5mm apart and sutured in place. The telemetry transmitter was placed into the abdominal cavity of the mouse. Perioperative analgesics were administrated at the onset of surgery (buprenorphine, 0.1mg/kg; meloxicam, 5mg/kg) and the next day (meloxicam, 5mg/kg). Saline (0.9%, 500µl) was also administered by subcutaneous injection at the end of the surgery. After surgery, the animals were allowed to recover for more than 2 weeks and 24 h EEG data-collection began when the mice were 20.1 ± 0.4 (mean ± S.E.M.; n=4) weeks of age. The telemetric transmitters were activated 1-2 days before recordings, and EEG/EMG signals were then recorded continuously (Dataquest ART; DSI). The EEG and EMG signals were modulated with a one pole high-pass (-1.1dB at 1.0Hz; -3.8dB at 0.5Hz) and a two pole low-pass antialiasing (-1.6dB at 50Hz) analogue filters built in the transmitter. An additional (30th order low-pass FIR) digital filter was selected at 49Hz (2dB of attenuation). These signals were visually classified into 10s epochs of vigilance states according to standard criteria ^22^.

### Statistics and figures

**Figure 1.**
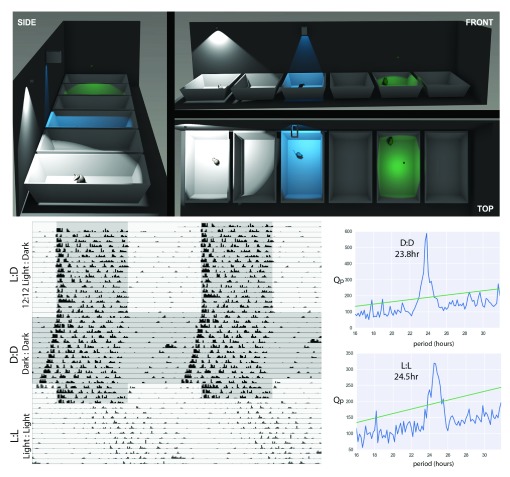
Considerations for a system for monitoring activity and rest. (Top Panel) Positioning of sensors and shielding to ensure accuracy of readings: Positioning of the sensors and any required shielding can be calculated using the angle of detection for any given sensor. In the case above, a sensor positioned 440mm above the cage floor at the back of the cage (WHITE) will also sense movement in neighbouring cages. This can be prevented by shielding the sensor (BLUE), or moving the sensor centrally, 220mm above the floor (GREEN). At different heights the sensor will be activated by different degrees of motion. Models created in the Blender 3D modelling program (version 2.68a, http://www.blender.org/) with each PIR represented as a lamp with the field of illumination the same as the field of detection for the PIR. Interspersed cages in the illustration are left empty to aid with clarity. Accurate positioning of the sensors will negate cross-talk and remove the need for empty cages.; (lower panel) Examples of actograms collected from a single C57BL/6J mouse under a 12h:12h light:dark-cycle, constant darkness and constant light, with Chi-squared periodograms showing the main rhythmic component of activity under these 2 constant conditions (sensor in equivalent position to blue light in top panel).

Correlation coefficients (Pearson’s r) were calculated and correlation plots and hierarchical clustering produced using the Seaborn package for Python ( http://stanford.edu/~mwaskom/software/seaborn/, version 0.7.0). Actograms and Chi-squared periodograms in Figure 1 were generated using the ActogramJ plugin (version 0.9-1.0, http://actogramj.neurofly.de/) ^23^ for the ImageJ program (version 1.5.1g, http://imagej.nih.gov/ij/), and based on 10min bins of mean activity to improve clarity. Other figures were generated using Matplotlib ^24^ ( http://matplotlib.org/, 1.5.1) and final figures were arranged using Adobe Illustrator and Photoshop (CS5, Adobe Systems Inc.)

## Results

PIRs with inbuilt amplification and a binary digital output, used alongside a light-dependent resistor (LDR) provide the simplest configuration that could be used for consistent measurement of activity. Whilst LDRs provide a good marker for gross changes in the light environment, this is no replacement for a correctly measured and calibrated light source, especially when considering circadian and visual processes ^25^. The experimental setups are shown visually in Figure 1 and further details of the experimental setup can be found in the Supplementary materials.

### Circadian measures of activity can be established using PIRs

Studies initially ensured that no cross-talk between cages was occurring, before characterising the degree of motion that was required for activation of the PIR sensor. The sensitivity of the detectors is dependent on the lensing and the distance of the sensor from the cage floor. Using information about the angle of detection (in this case 91°) the field of detection can either be calculated, or the environment modelled using 3D-modelling software (Blender, version 2.68, http://www.blender.org/), see Figure 1, **upper panel**). This approach is useful when considering the environment in different animal facilities to show the options for positioning or shielding sensors, to prevent cross-talk. PIRs were then incorporated into the microcontroller system, where data could be analysed using existing tools ^23^. This demonstrated circadian entrainment, with elevated nocturnal activity and clear activity onsets, free-running activity in constant dark (DD) with a circadian period of <24h and period-lengthening in constant light (LL) with a circadian period >24h ( Figure 1, **lower panel**). For comparison, these PIR sensors were also incorporated into an established system for studying circadian behaviour (ClockLab, Actimetrics, IL), providing comparable results.

To determine the activity threshold required to activate the PIR, video of mice in cages was recorded, while the output of the PIR was linked to a circuit including a near-infrared LED (emission peak 850nm). This LED was placed in the corner of the field of view of the camera, outside and below the top of the cage. As sensitivity is determined by how close the PIR was positioned relative to the cage floor, this was assessed under two PIR positions 440mm and 220mm (equivalent to BLUE and GREEN positions in Figure 1, **top**). At 440mm, activation every 1–2 steps was observed (gross locomotion), whereas at 220mm, activation of the PIR was also observed with small movements, such as head turns and rearing (see Video). This ability to separate small movements from immobility raised the possibility that this approach could be used to identify sleep in addition to locomotor activity ^13, 15^.

### Sleep scored by immobility under PIR correlates well with total EEG-scored sleep

**Figure 2.**
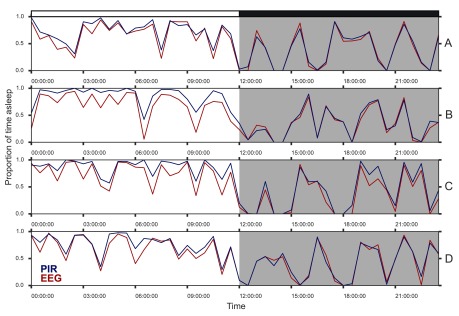
Sleep, as assessed by EEG recordings and by immobility. Comparison of periods of immobility (> 40s) measured by PIR to manually-scored sleep from EEG telemetry (as proportion of 30min bins), in 4 male C57BL/6J mice, entrained to a 12h:12h light:dark-cycle (labelled A to D).

**Figure 3.**
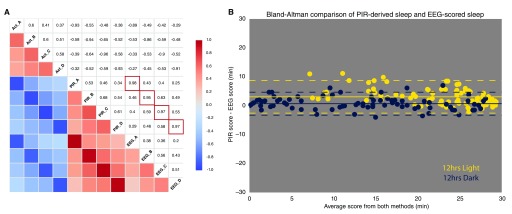
Immobility as a correlate of sleep, and compared to total sleep scored from EEG/EMG records. **a**) Correlation plot shows the relationships between the activities of 4 C57Bl/6J mice, the sleep scored from periods of immobility between bouts of activity and the total sleep scored by EEG/EMG in the same mice. High correlation between PIR and EEG: Pearson correlation coefficients of 0.95-0.98 for sleep per 30min bin (scored by immobility >40sec by PIR and by manual scoring of total sleep by EEG traces, red boxes). **b**) Bland-Altman comparison of methods for scoring total amounts of sleep. Good agreement of the total sleep in each 30min bin is achieved in the 12hrs of dark (PIR-EEG = +0.7min, -3.3 to +4.7min 95% C.I.). As is apparent from the individual traces (see Figure 2) the agreement is lower in the 12hrs of light (PIR-EEG = +3.1min, -2.6 to +8.7min 95% C.I.). This is likely to the reduced overall movement in the light leading to over-estimation of total sleep by the PIRs. A closer look at the data from Figure 2, with 5min bins for sleep as measured by EEG and PIR, supports the suggestion that bouts of quiet wakefulness (with minimal or no associated movement) would be detected by EEG but may be missed in any measure of sleep based on extended immobility (see File 2_supplementary). Although these bouts of waking make minimal difference to overall measurement of time asleep, they can lead to longer bouts of sleep being reported.

Using a criteria of >40sec of immobility as used previously ^13, 15^, a PIR at 220mm above a cage floor was found to provide a high correlation with sleep assessed by video-tracking. As such, PIR-determined sleep was directly compared against sleep measured using EEG/EMG in the same animals. C57BL/6 mice (n=4) were implanted with dual biopotential telemeters (DSI, St. Paul, MN, USA). Following recovery and entrainment to a 12:12 light:dark-cycle, 24h of activity from PIRs at 220mm above the cage floor was compared to EEG/EMG scored sleep ( Figure 2). A high degree of correlation was observed with the total amount of sleep reported by EEG/EMG (>0.95 in all mice, Pearson’s r, Figure 3, **left**). A Bland-Altman comparison of methods ^26^ showed a good agreement of the total sleep in each 30min bin (PIR-EEG = +1.9min, -3.5 to +7.3min, 95% confidence intervals), with a slightly lower agreement in the 12h of light ( Figure 3, **right**). This is likely to be due to reduced overall movement in the light leading to over-estimation of total sleep by the PIRs as has been reported for other immobility-based methods ^15^.

### Long-term monitoring of activity and immobility-scored sleep

**Figure 4.**
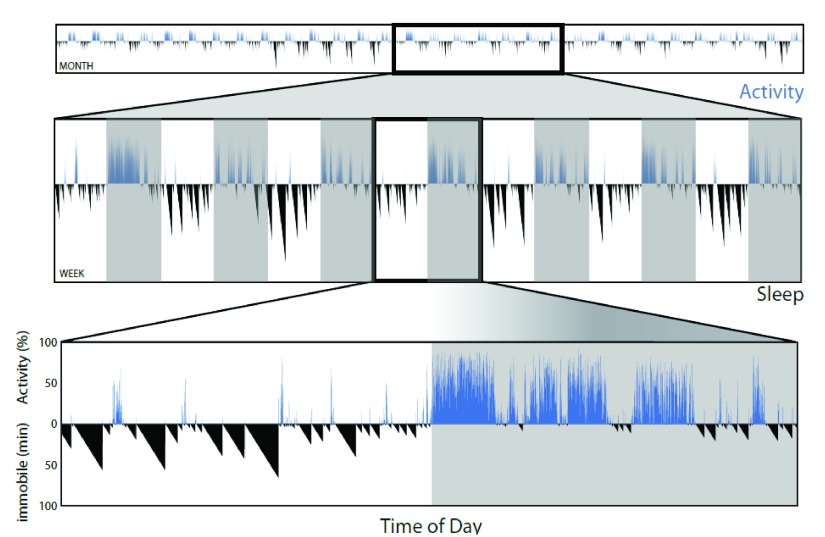
Minimal data processing allows for long-term longitudinal studies of patterns in both activity and sleep. Traces for activity (blue, upward deflection) and sleep (black, downward increasing deflection in minutes for immobility longer than 40 seconds) from a single male C57BL/6J mouse for 1 month. Lower panels show one week (middle), or a 24h period lower panel) from within this data at higher resolution.

Removing the need to collect and process video files allows the assessment of activity and sleep (as immobility over 40s) over longer periods of time. Figure 4 shows an example of the length and detail of recording that is possible. A time series of activity data (and from immobility, sleep) over 1 month can be examined in more detail in sections of 1 week, or a single day.

### Wild-type laboratory mice show repeatable patterns of activity from day to day, but these patterns differ between individuals

**Figure 5.**
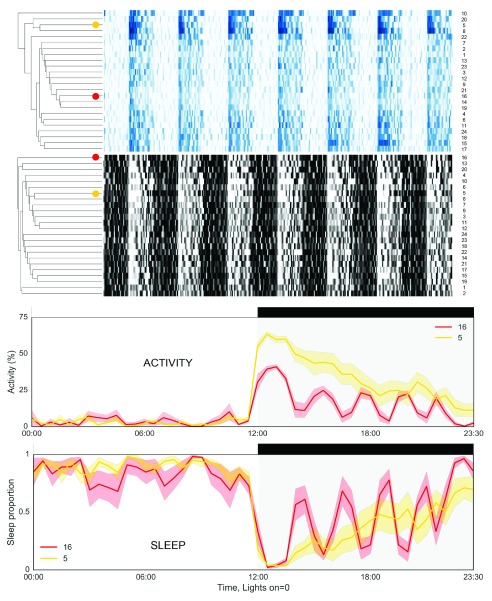
Variation in the daily patterns of activity and sleep between wild-type mice. Hierarchical clustering of activity (blue, top) and sleep (black, bottom), for 24 male wild-type C57BL/6J mice shows a range of different patterns. Lower graphs indicate that from day to day each mouse repeats a similar pattern over each 24h cycle (lower intra-day variation). However, variation in amplitude and distribution of activity and sleep can be much greater between mice. Example traces (taken from the marked positions on the heatmap above) show mean activity (for 7 days) for a single male mouse in 30min bins across the day, with shaded areas showing the standard error of the mean (S.E.M.).

When studying any biological process, rhythmic variation in measurements is often important but either ignored or not studied. Attempts to study variation over time will usually involve collecting time series of data. These can either be longitudinal (multiple measurements within the same subject) or transverse (a time series constructed from measurements from many individuals). The data in Figure 5 reveals the importance of longitudinal measurements where possible. Unsupervised hierarchical clustering of 1 week of data from 24 male wild-type mice shows that although many animals show repeating patterns of activity from day to day (high interday stability), these patterns of activity and sleep are often different to those mice in neighbouring cages. Clusters do not just consist of those mice in the same environmental chamber (4 chambers, with mice numbered 1–6, 7–12, 13–18 or 19–24 housed together, Figure 5). The clustering also indicates the measure of sleep from immobility does not result in the same clustering as activity, showing that sleep is not just the absence of activity and that cycles of activity and sleep are distinct (yet related) biological processes. Furthermore, differences in the positions of individual mice in the heatmaps of all immobility (>0s) vs sleep defined as extended immobility (>40s) show that, although the estimation of sleep is related to immobility, use of immobility (>0s) alone will overestimate sleep. Moreover, the relationship between these two measurements is not the same for all individuals ( File 5_supplementary). Longitudinal measurement over multiple days also reveals additional ultradian rhythms in activity and sleep in some mice ( Figure 5, **lower panels**).

Examples of movements of a mouse required to activate PIR sensors at different heights from the cage floor Near-infrared LED outside and below the cage shows the activation of the sensor. Click here for additional data file. Copyright: © 2017 Brown LA et al. 2017 Data associated with the article are available under the terms of the Creative Commons Zero "No rights reserved" data waiver (CC0 1.0 Public domain dedication).

## Discussion

Here we have shown that passive infrared sensors (PIRs) coupled with an LDR and a readily available microprocessor provide a simple, affordable system for the combined measurement of activity, light and sleep over days, weeks, or even months ( Figure 4). We have named this system COMPASS - Continuous Open Mouse Phenotyping of Activity and Sleep Status. COMPASS enables researchers working on transgenic mouse models in any discipline to easily use activity and sleep as biomarkers without the need for specialist resources or expertise. Longitudinal monitoring of activity and sleep will also help to assess the effect of any novel therapeutic interventions in pre-clinical models and act as potential objective welfare indicators. Early incarnations of the system have already proved valuable for studies of a number of mouse models of disease ^27, 28^, with phenotypes described using video-based assessment of sleep and the COMPASS system appearing the same (both offering an important comparison to patterns of wheel-running behaviour) ^29^. Measures of immobility will potentially have difficulty detecting phenotypes such as narcolepsy, if the onset of sleep is sudden and sleep duration is short. However, as recent studies of orexin knockout mice with a non-invasive piezo-electric system to assess have suggested, the total daily amount of sleep between wild-type and transgenic animals was the same, suggesting that missed bouts of narcoleptic sleep (occurring with without preceding inactivity), may lead to different total amounts of sleep being recorded ^30^. As always, users should always be mindful of the temporal resolution and assumptions in the measurements they employ.

There are a number of labs working to explore non-invasive methods of assessing sleep and both video-tracking and piezo-electric systems have proved to be valuable in the study of sleep in mutant mouse models of disorders such as narcolepsy ^30^ and Huntington’s disease ^31^. Furthermore, modelling and machine learning using EEG/EMG data ^32^, video-tracking ^33^, or piezo-electric data ^34^ are helping to improve the scoring of different stages of sleep, especially for Rapid Eye Movement (REM) sleep. There are also systems for monitoring the behaviour of multiple co-housed animals, including patterns of activity and rest ^35^. COMPASS prioritises simplicity and efficiency for assessing sleep, while additionally providing a measure of locomotor activity. Although this system is simple, this allows both the cost (from around £20 per cage) and size of the data (1-2Mb per cage per month) to be kept to a minimum. Affordability and minimal data requirements mean such a system is ideally suited for web-based data logging, enabling researchers to monitor their ongoing experimental animals remotely and the open nature should allow for integration of other environmental and physiological sensors.

It is important to note that PIRs will only measure total movement in a cage, necessitating single-housing to establish individual patterns of activity and sleep. Although this is the standard experimental approach used in circadian and sleep laboratories, group housing is preferable for long-term colony housing. However, COMPASS is also suitable for monitoring total cage activity in group housed mice, where altered behavioural patterns or health status in one animal may be identified via changes in whole cage activity. Further studies may be required to establish standards for group monitoring in this manner. Non-invasive, longitudinal measurement of both activity and sleep status would be transformative for many fields of research, as well as animal welfare. Recent studies have shown that, in models relevant to psychosis, depressive and anxious states are correlated to infradian variation in home cage locomotion ^36^. Furthermore, recent work shows that the success of haemopoietic stem cells transplants in repairing blood and bone in a recipient mouse are abrogated when the donor is sleep deprived ^37^. This suggests prior activity and sleep history are important variables in many if not all animal experiments, ones that are currently often ignored. This study details an important step towards the goal of widespread longitudinal measurements of activity and sleep in laboratory animals.

## Data and software availability

The data referenced by this article are under copyright with the following copyright statement: Copyright: © 2017 Brown LA et al.

Data associated with the article are available under the terms of the Creative Commons Zero "No rights reserved" data waiver (CC0 1.0 Public domain dedication). 

The programs (sketches) for the Arduino microcontroller and data-collection (via Processing) are provided online ( current GitHub repository), alongside a series of interactive Python notebooks ( http://jupyter.org/). All PIR data for assessing activity and sleep in mice is also provided in this repository ( Figure 2– Figure 5), as is the sleep scoring from the EEG data used for comparison in Figure 2 and Figure 3.

Zenodo: Dataset 1. The programs for hardware, Python notebooks, and files from Dataset 2, 10.5281/zenodo.345396 ^38^

Zenodo: Dataset 2. PIR data, and sleep as scored from EEG files (.csv), 10.5281/zenodo.160344 ^39^

Zenodo: Dataset 3. The raw EEG data, 4 files (EEG_A to D), in European data format (.edf), 10.5281/zenodo.160118 ^40^

*Figshare:* Examples of movements of a mouse required to activate PIR sensors at different heights from the cage floor. doi: 10.6084/m9.figshare.4072701 ^41^

## Supplementary material

**Supplementary File 1. Additional details for construction of a 6-channel system.**

.

**Supplementary File 2. Sleep, as assessed by EEG recordings and by immobility (5min averages).**

Comparison of periods of immobility (> 40s) measured by PIR to manually-scored sleep from EEG telemetry (as proportion of 5min bins), in 4 male C57BL/6J mice, entrained to a 12h:12h light:dark-cycle (labelled A to D).

**Supplementary File 3. Fritzing circuit board design for sensors.**

**Supplementary File 4. Fritzing circuit board design for microcontroller.**

**Supplementary File 5. Variation in the daily patterns of inactivity and sleep compared.**

Hierarchical clustering of inactivity (red, top) and sleep, as defined by ≥ 40s in such immobility (black, bottom), for 24 male wild-type C57BL/6J mice. Differences in the positions of individual mice in the heatmaps show that, although the estimation of sleep is closely tied to overall immobility, the relationship between these two measurements is not the same for all individuals.
