# Supplementary material for: COMPASS: Continuous Open Mouse Phenotyping of Activity and Sleep Status
Source: Wellcome Open Res. 2017 Apr 24;1:2. Originally published 2016 Nov 15. [Version 2] doi: 10.12688/wellcomeopenres.9892.2 (PMC5140024; doi:10.12688/wellcomeopenres.9892.2)
Supplement: Supplementary file 2 [file wellcomeopenres-1-11937-s0001.tgz › f012be9c-147b-48a6-818d-01cf1989e5dd.pdf]

Supplementary material

Building a system for monitoring activity:

Choice of microcontrollers

The scenarios included in the paper have all been run using the Arduino Uno (Rev3) board, featuring an ATmega328 microcontroller (http://arduino.cc/en/Main/ArduinoBoardUno). The capabilities of the boards in a laboratory environment have been covered in detail and the Arduino community provides a wealth of advice and support, beginning with detailed documentation (http://arduino.cc/en/Reference/HomePage and http://playground.arduino.cc/). The reference sketch used in the current study requires only 6 of the 14 digital input/output pins on the board and one of the 6 analog inputs. Even greater expansion of monitoring capabilities can be achieved using the Arduino Mega board.

There are also other architectures that may provide additional features, while still being programmable via an accessible development environment (such as the mbed hardware, http://mbed.org/). Although microcontrollers are ideal for simple sensing and reliable timing, they have little processing power. Combining input/output pins with a higher degree of computational power can be found in devices such as the BeagleBone board (http://beagleboard.org/). In addition, the recent release of the low-cost Raspberry Pi computer (http://www.raspberrypi.org/) has provided another option for activity monitoring. The Raspberry Pis possess input and output pins for sensors and would also work well in combination with microcontrollers.

Sensors and circuits

In the example scenarios below a single PIR (model: Panasonic EW AMN32111) is used alongside a light-dependent resistor (LDR). The use of a PIR with integrated digital amplifier and pre-made circuit-boards help to ensure consistency in the behaviour of the sensors.

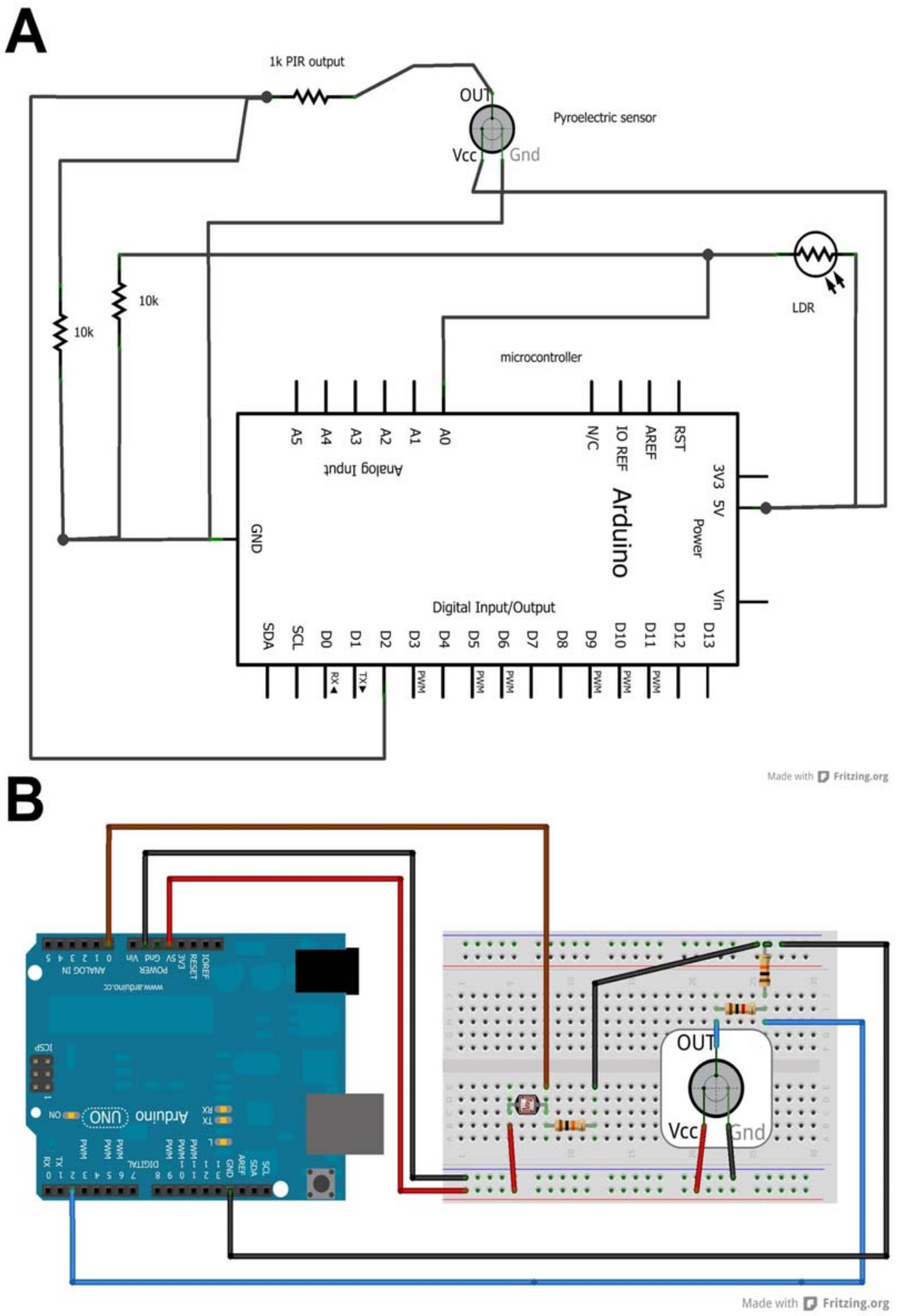

Supplementary Figure 1 *- An example of the most basic circuit used in the current study. A) Circuit diagram and B) breadboard-wiring diagram for a basic circuit linking a single PIR and light-dependant resistor (LDR) to an Arduino microcontroller.*

Supplementary table - Components and approximate costs for an activity-monitoring system.

Item	Cost (£)	for 6 channel system
Arduino Uno	16	16
Main enclosure	9	9
Pyroelectric motion sensor	10	60
Custom circuit board (sensor unit)	3	18
custom circuit board (main unit)	10	10
LDR Photocell	1	1
Resistors, headers and jumper cables	1	1
Connectors (RJ12) plus cable	2	12
ABS enclosure for sensor	0.5	3
USB cable	1	1
		£ 131.00
		Approx. £22 per channel
		(excluding PC)

Additional notes:

- Positions for 1k resistors and 10k resistors are clearly marked on the boards
- There is a space to solder either a 2-pin header or an LDR directly to any of the breakout boards for the PIRs. If using this option, solder and additional 10k resistor to that breakout board. The sketch for the Arduino is written to expect a single LDR, to be connected to analog pin2.
- Right-angled RJ12 connectors were used for the breakout boards for the PIRs, as this offers better options for positioning cables. Vertical RJ12 connectors were used on the base unit for the same reason.

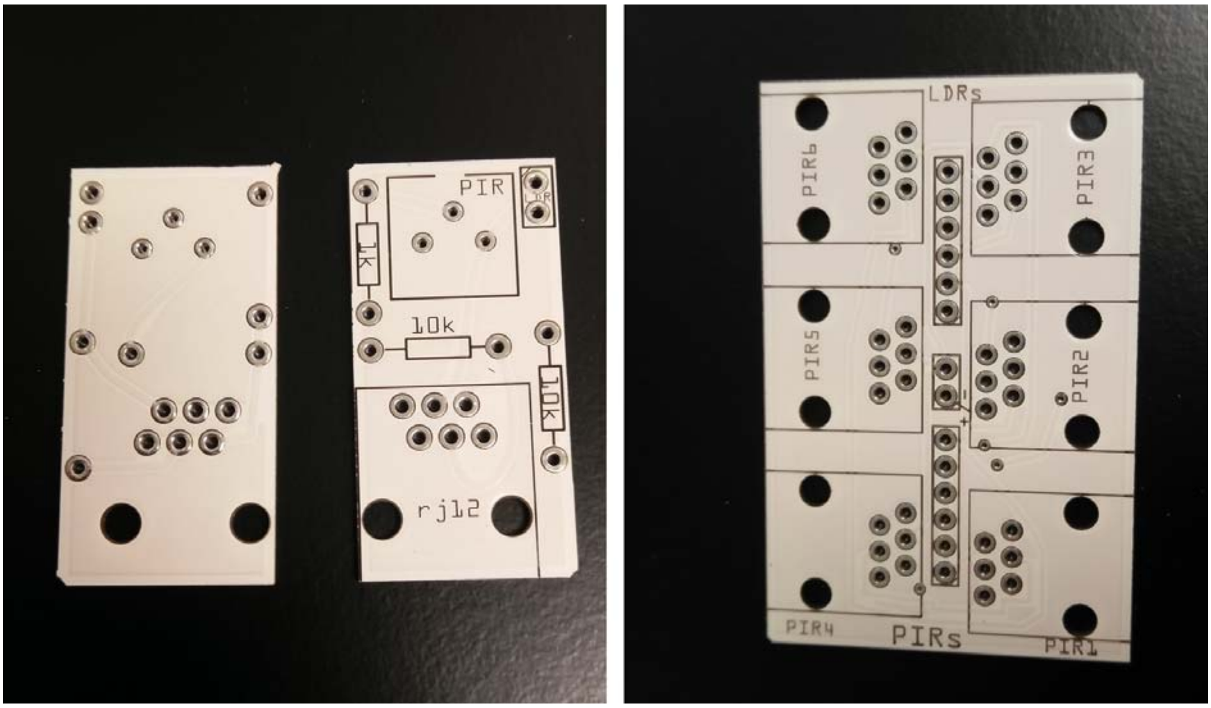

Supplementary Figure 2 – *Building the system. Above) Circuit boards before populating Below) completed PIR sensor, with and without casing (left) and wiring of base unit from the main board using male to male jumper cables(right).*

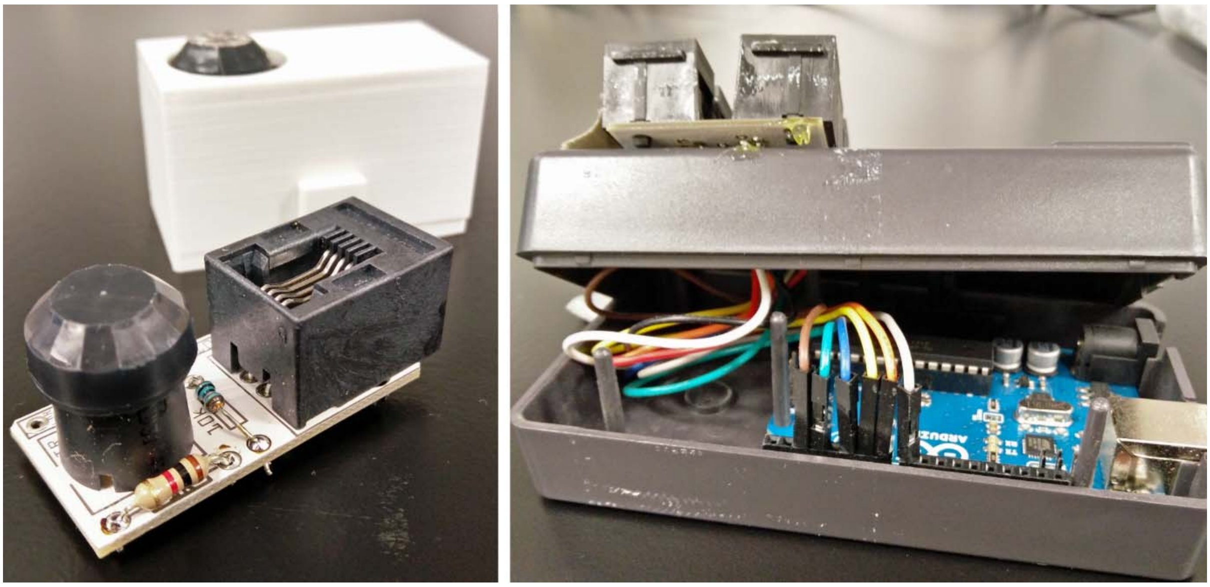

Cabling

For the current system we have used RJ12 cabling and connectors for joining the sensors back to the main board on the microcontroller. This provides a balance between cost, ease of use and performance. RJ12 cables can be bought pre-made or made to length easily. If following the reference design it is important to note that the cables should not be a

crossover type (Supplementary Figure 3). Unnecessary long cables should be avoided as these increase the chances of these acting as antennas for interference.

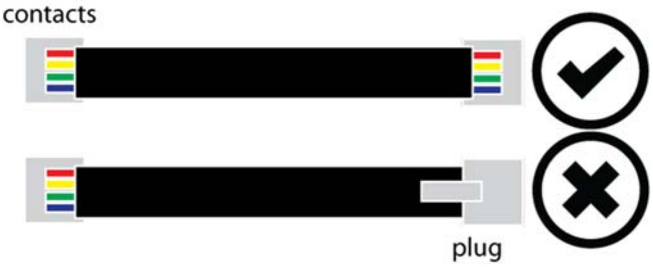

Supplementary Figure 3 – Order of coloured cores in cable should differ at each plug.

Testing:

Careful testing of the sensors when built is essential. This can be done in a number of steps:

- Following loading the microcontroller with the relevant sketch, sensors can be plugged in and readings collected from the >Tools >Serial Monitor heading of the Arduino IDE. Decreasing the inter-loop delay in the sketch will give faster feedback on when the sensors are active.
- Alternatively, the additional sketch provided as part of the paper can be used to light and LED when the sensor is active. Using a visible-wavelength LED provides a simple circuit for checking sensors.
- Following positioning of the system it is essential to record over empty cages to ensure all sensors are stable and consistently reading zero. Introducing animals to cages under every other PIR will ensure that recordings are being obtained while neighbouring sensors remain inactive. Move cages to confirm the same is true for the remaining sensors. This process should be repeated before all new experiments.

Computing requirements:

The system has minimal computational requirements and has been successfully run on old computers (< 512Mb RAM, and <1GHz Pentium III processors), and operating systems from Windows XP- Windows10 (Microsoft, Redmond, USA), MacOS X (Apple Inc., USA) and Ubuntu Linux 14.04 LTS (Canonical Ltd.)

Software and the collection of data

The code for this first example is provided as two components. The first is the code that is uploaded to the microcontroller itself. As the comments show, the code first defines how

the microcontroller communicates with the computer connected via the USB port and to which pins the PIR and LDR are connected. The program then repeats a loop where the state of the PIR and the LDR are read and the readings sent to the computer, preceded by a header as comma-separated values.

Extensive documentation exists to help newcomers begin to work with to microcontrollers and the Arduino ecosystem in particular. The sketches (programs) provided have been tested with versions 1.0.1-1.0.5 and 1.5.6- 1.6.11 of the Arduino integrated development environment (IDE, available at http://arduino.cc/en/Main/Software)

The corresponding program (sketch) in Processing can be opened and run after the Arduino IDE is closed (otherwise they will compete for access to the USB device. This software (Processing) is also open-source and available for multiple platforms (see http://processing.org/). The programs detailed in the current paper work with version 2.1/2.2.1

When loaded and played, this program will a read the serial messages sent by the Arduino and do three things:

- Check the time the message was received
- Append the time and values to a comma-separated text file (.csv file) created at the beginning of the program
- Display the data on screen as a histogram

The text file is created with a name corresponding to the time the device starts recording. This type of file can then easily be imported into any spreadsheet for analysis, or used in conjunction with free software for circadian analysis, such as the ImageJ plugin, ActogramJ (http://actogramj.neurofly.de/). It is worth noting that ActogramJ does not handle gaps in data and needs careful checking to ensure the correct columns and rows are defined for data. Additionally, there is no awareness of date and time data, so the correct number of time points per day needs to be provided (24 for hourly averages, 144, for 10min, 1440 for 1min, 8640 for 10s).

Timing:

The data in the current study can be further optimised so that the total time to collect, send and record each message is exactly 10 seconds. Once complete, the actual time between data can be established from the timestamps in the '.csv' files. In the Arduino sketch, changing the delay between each of the 100 loops and adding a further short delay before sending the message can be used to create a millisecond-accurate gap between serial messages. However, all the data in the current paper shows the system will accurately report activity and sleep without any further adjustment.

Common issues:

1. Not acquiring the Arduino microcontroller board after plugging in:
    a. Check Arduino IDE is open.
    b. Attach board by USB cable
    c. Check >Tools menu for correct >board and >port entries
    d. If this fails, unplug microcontroller, and plugin again.
2. No readout or file saved after running the sketch in Processing:
    a. Close Arduino IDE
    b. Run Processing Sketch again
    c. The program puts out a list of available serial ports at start up (in black panel at the bottom), with their assigned indices.
    d. If the histogram window does not appear, the program will be looking for incoming messages from the wrong port. This is solved by changing the 'portIndex1 =' value towards the top of the processing sketch and trying again.
3. Unexpected readings:
    a. Floating random values (ungrounded pin, normal if not sensor is connected).
        i. Check cable and sensor (try new ones). Incomplete circuits, usually due to soldering errors.
        ii. Check connections on the main board and that the board is connected correctly to the pins on the Arduino microcontroller.
    b. Constant readings of '0' or '100' :
        i. If on all sensors, problem is probably with main board or microcontroller (disconnect all cables one by one).
        ii. Ensure RJ12 cable does not cross-over (see cables section)
        iii. On single sensors replace cable and or sensor until problem is identified. If still faulty check connector on main board and jumper wire from main board to microcontroller.
    c. Problems with reading environmental light:
        i. Pointing the face of the LDR towards the light source can improve the range of values in light and darkness
        ii. Ensure legs of the LDR are not crossed and touching. This will short circuit (bypass) the resistor leading to constant readings of 0.
