# Supplementary figures and images for: COMPASS: Continuous Open Mouse Phenotyping of Activity and Sleep Status
Source: Wellcome Open Res. 2017 Apr 24;1:2. Originally published 2016 Nov 15. [Version 2] doi: 10.12688/wellcomeopenres.9892.2 (PMC5140024; doi:10.12688/wellcomeopenres.9892.2)

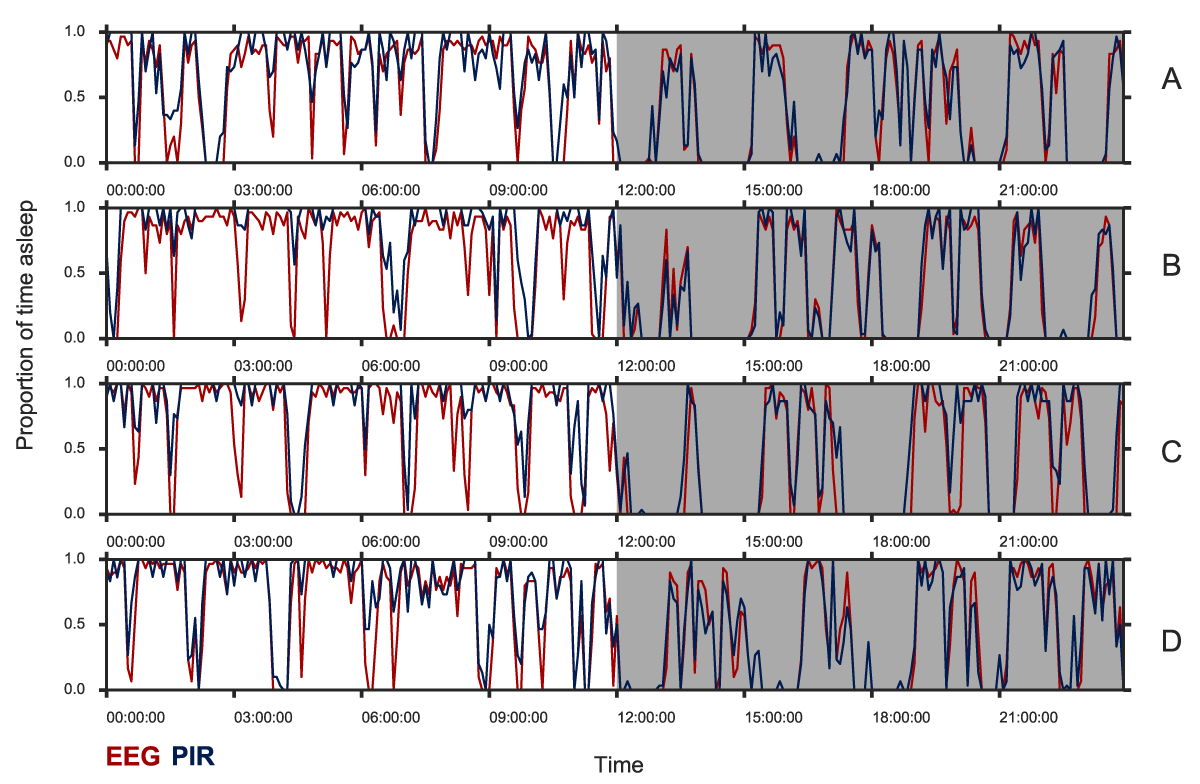

Supplement: Supplementary file 3 [file wellcomeopenres-1-11937-s0004.tgz › f4f7c056-1baf-42c2-8bd9-41cf7966bcb4.png]

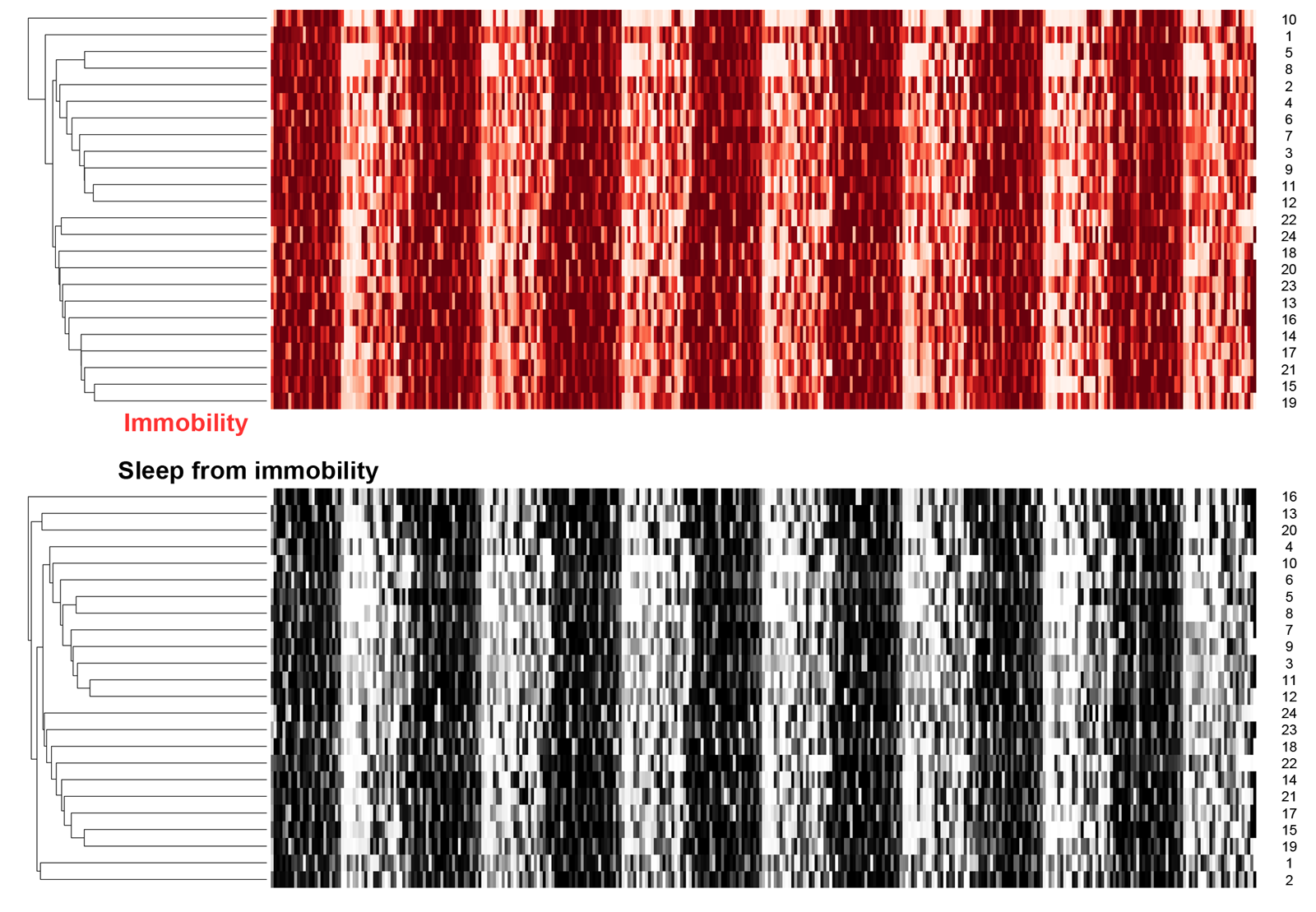

Supplement: Supplementary file 6 [file wellcomeopenres-1-11937-s0005.tgz › ae18756c-9312-4a49-b80f-65273ad188a7.png]
