# Supplementary material for: COMPASS: Continuous Open Mouse Phenotyping of Activity and Sleep Status
Source: Wellcome Open Res. 2017 Apr 24;1:2. Originally published 2016 Nov 15. [Version 2] doi: 10.12688/wellcomeopenres.9892.2 (PMC5140024; doi:10.12688/wellcomeopenres.9892.2)
Supplement: Peer review history

# Peer review: Referee response for version 2

**DOI:** 10.21956/wellcomeopenres.11937.r22134

**Published:** 2017-05-03

**Authors:** Christopher Colwell, Dawn Loh

**Affiliations:** 1 Department of Psychiatry and Biobehavioral Sciences, University of California - Los Angeles, Los Angeles, CA, USA

I am satisfied with the response and feel that this work will make a solid contribution to this research area.

We have read this submission. We believe that we have an appropriate level of expertise to confirm that it is of an acceptable scientific standard.

# Peer review: Referee response for version 1

**DOI:** 10.21956/wellcomeopenres.10663.r17584

**Published:** 2016-12-09

**Authors:** Hugh Piggins

**Affiliations:** 1 Division of Diabetes, Endocrinology & Gastroenterology, University of Manchester, Manchester, UK

In this manuscript, Brown and colleagues report a new, low cost (COMPASS) system based on passive infrared detectors (PIR) to longitudinally assess sleep in mice.  This is an important advance since conventional methodologies to measure sleep  in rodents are invasive and require considerable finances and infrastructure. Further, such approaches result in the acquisition of large volumes of data whose analyses are complex and time consuming.  The quality and quantity of sleep is widely recognized as important for general health across a wide range of organisms and method described in this manuscript provides an interesting and readily adaptable system to enable many labs to quantify sleep in mice. This approach quantifies immobility (40s epochs) and uses this as an index of sleep state.  They validate COMPASS through careful and sophisticated comparison to sleep as measured through the conventional electroencephalogram (EEG) method.  In general this is a very well-written and nicely illustrated manuscript, with the details and validation of COMPASS readily understandable.  

  

1. In the analysis of this PIR-based method, mice with fully functional circadian and sleep regulation were used (the C57BL6 animal).  Can the authors speculate on the effectiveness of this approach in animals such as orexin knockout mice in which overall sleep in 24h does not differ to that of controls, but where there is an increase in cataplexy? Specifically, how will this method distinguish between immobility due to a motor disorder versus a bonafide sleep alteration?

I have read this submission. I believe that I have an appropriate level of expertise to confirm that it is of an acceptable scientific standard.

# Response

**Published:** 2017-04-11

**Authors:** Laurence Brown

**Affiliations:** University of Oxford, UK

Dear Professor Piggins,

 Thank you for the helpful review of our work.  We have added to the discussion in order to stress the importance of careful interpretation of results for sleep based on immobility alone.  It is interesting that sudden-onset bouts of sleep (cataplexy) might be missed or underestimated using ≥40s of immobility as criteria, but that in groups with the same overall amounts of sleep, this would lead to a decrease in the reported sleep each day and therefore a difference when compared to wild-type litter-mates. 

 Our recent use of the COMPASS system alongside voluntary running wheel activity would suggest that the PIRs often give a more sensitive and unbiased measure of overall activity. Mice are often awake active, but not running in a wheel. However, we acknowledge that more data is needed before activity (and consequently, immobility) alone could be used to reliably separate motor deficits from changes in the parameters of circadian rhythmicity or sleep, especially as many of the mutant mice studied have complex phenotypes

# Peer review: Referee response for version 1

**DOI:** 10.21956/wellcomeopenres.10663.r17583

**Published:** 2016-12-06

**Authors:** Christopher Colwell, Dawn Loh

**Affiliations:** 1 Department of Psychiatry and Biobehavioral Sciences, University of California - Los Angeles, Los Angeles, CA, USA

There is a large interest in the role of sleep in our health and in the disease process.  One of the major limitations in the study of sleep is the difficulty in measuring sleep using  electroencephalography (EEG) with the required surgical procedures and time-consuming data analysis.  The Peirson group has previously made good progress using a video based system to quantify sleep behavior.  This system has been used by laboratories around the world.  So we read with interest this study which describes simple, non-invasive system to measure home cage activity in mice based upon passive infrared (PIR) motion sensors. Careful calibration of this system will allow users to simultaneously assess sleep status in mice. The use of open-source tools and simple sensors keeps the cost and the size of data-files down.  We looked over the plans and felt that we could implement this system.  Broadly, this is a very promising approach to address an important issue i.e. measurement of sleep in rodents using a high-throughput system. 

 While we are generally supportive, we did have some problems with the present ms.  First, as the authors well know, inactivity is not the same as sleep.  Some more validation of their sleep measurements is important.  For example, do hypnotics increase and stimulants alter PIR defined sleep?  When the mice are sleeping can they demonstrate the increase in threshold to external stimuli?  The authors undertook this type of validation in their prior study with video analysis and we feel that they should do the same type of validation with this new methodology. 

 In addition, we would like them to compare sleep fragmentation with the IR and compare that to values obtained with the EEG.  Sleep fragmentation is an important parameter in disease models and should be detected with their methodology.  Their prior video-based system was effective in measuring this parameter. 

 It was unclear if the mice needed to have an empty cage in between the measurement cages.  We do not see why this would be the case but the illustration in Fig. 1 suggests the need for a separating cage.  Please clarify. 

 We were uncertain as to the utility of the cluster analysis.  We did like the clear demonstration that the intra-day patterns were stable between mice.  But we felt that the cluster analysis would be more useful if it compared the inverse of activity in top panel with the IR-defined sleep below.  This would help address the issue of whether IR-defined sleep is more than the absence of activity. 

 Finally, several labs have been exploring the use of behavior measures of sleep including IR measures as used in the present study.  The authors should expand this part of their discuss to include others working in this space.

We have read this submission. We believe that we have an appropriate level of expertise to confirm that it is of an acceptable scientific standard, however we have significant reservations, as outlined above.

# Response

**Published:** 2017-04-11

**Authors:** Laurence Brown

**Affiliations:** University of Oxford, UK

Dear Dr Loh and Professor Colwell

 Thank you for your review and the important points that you raise. On the first point we agree that we should continually reassess of the use of immobility as a surrogate of EEG-defined sleep. However, we would consider the use of passive infrared sensors as a more efficient way of assessing immobility, rather than a new methodology to measure sleep.  As you have discussed, the previous work from the group with video tracking showed that hypnotics increase and stimulants decrease immobility.  It is definitely the case that data from the COMPASS system will allow efficient measurement of bouts of both activity and sleep, as well as measures of stability in these bouts.  This is one area where long-term recordings will be especially useful.  We are focusing current and future efforts on this aspect of analysis, but we wish to collect data from a number of known sleep and circadian mutant models first. One additional advantage of the .csv file format used is that it provides easier import of data in to existing programs for bout-analysis and measures of fragmentation with which groups might already be familiar.  In order to help demonstrate how analysis of bouts could be approached we have included another notebook for download with the revised paper (COMPASS_sleep_bouts.ipynb).

 It is also possible to look at the PIR and EEG-defined sleep in **Figure 2** at a higher temporal resolution. Averaging sleep with each system over 5min bins further supports the suggestion that small waking movements in the light period would be the main source of error for the PIR-based estimate of sleep.  Consequently, this would lead to longer uninterrupted bouts of sleep being recorded with PIRs. A note to this effect has been added to the revised manuscript (along with a supplementary version of **Figure 2**).

 You are correct that, provided the sensors have been correctly tested, there is no need to leave empty cages between those in use.  As one sensor is shown reading from a neighbouring cage, we left some empty in **Figure 1**.  A note to this effect has been added to the legend of **Figure 1**.

 We have added a comparison of the hierarchical clustering of the 24 wild-type mice from immobility (>0s) verses sleep defined as extended immobility (>40s)  (now included in the revised paper as a supplement to **Figure 5**). This shows that sleep will be over-estimated based on immobility alone, and that the clustering of mice by their immobility (>0s) vs extended immobility (>40s) profiles differs, illustrating that sleep behaviour is not simply the inverse of locomotor activity.

  Finally, we agree that by keeping the focus on the practicalities of this particular system, we neglected to describe the extent of other efforts towards a non-invasive estimate of sleep.  We have expanded the discussion in an attempt to cover others working in this field, with regard to both improvements in methodology and uses of non-invasive measures of sleep in biomedical research.

# Peer review: Referee response for version 1

**DOI:** 10.21956/wellcomeopenres.10663.r17581

**Published:** 2016-12-01

**Authors:** Valter Tucci

**Affiliations:** 1 Neuroscience and Brain Technology Department (NBT), Istituto Italiano di Tecnologia (IIT), Genoa, Italy

In the manuscript ‘COMPASS: Continuous Open Mouse Phenotyping of Activity and Sleep Status’, Brown *et al.* describe the development of a new method to approach the investigation of sleep in mice. In particular, by using passive infrared sensors the system recapitulates the main distribution of physiological sleep in animals. This is a very important study in the field of sleep, in which EEG time-consuming approaches are currently still the standard. The authors have provided a comprehensive evaluation of the PIR based system with EEG-based (10-sec epochs) sleep scoring, the manuscript is well written and the methods precise, as well as results and discussion. I have no major issues to raise but only two minor questions that perhaps would be useful to clarify.

- The authors report a high correlation between PIR at 220nm and sleep. If I understand correctly this was better than the PIR at 440nm. Was this a significant improvement?
- The main comparison reported in the paper is between PIR detected immobility and EEG. However, at Page 6 second column, the authors discuss of a high correlation with video-tracking. Was this paralleled with EEG or on different mice?

I have read this submission. I believe that I have an appropriate level of expertise to confirm that it is of an acceptable scientific standard.

# Response

**Published:** 2017-04-11

**Authors:** Laurence Brown

**Affiliations:** University of Oxford, UK

Dear Professor Tucci,

 Thank you for your time and effort reviewing the work. In answer to the points you raise we can confirm that the original positioning of the PIR sensor at 440mm was at the top of our environmental chambers, in which the cages sat.  As is indicated by the video we published, the detection of small movements by the mice was poor at this height.  Our findings in preliminary experiments looked at sleep assessed by the video-tracking protocol previously used in our lab and the PIRs. We found the correlation of video-tracking and PIRs at 440mm from the cage floor often fell below 0.75 (Pearson’s r, over 24h), but with the sensors at 200-220mm from the cage floor, the correlation between methods was very high (Pearson’s r, >0.98).  These experiment were carried out on different mice to the EEG experiments.  As both of these methods are based on immobility we felt it was best to focus on the comparison with EEG-scored sleep.

# Peer review: Referee response for version 1

**DOI:** 10.21956/wellcomeopenres.10663.r17579

**Published:** 2016-11-22

**Authors:** Dominic Wells

**Affiliations:** 1 Department of Comparative Biomedical Sciences, Royal Veterinary College, London, UK

This is an excellent piece of work describing a simple, low-cost, non-invasive system to measure mouse activity in their home cage. The system is based on passive infrared (PIR) motion detectors. Using 40 seconds of immobility as an indicator of sleep, the authors demonstrate that PIR based estimates of sleep correlated very well with EEG recordings from implanted telemetry transmitters.

 In general, the work is very well described, allowing other researchers to use this system in their animal facilities, but there are a few points that it would be useful to clarify:

1. Second paragraph of the introduction. The fourth sentence should be rewritten to make it clear that the import, re-derivation and breeding issues relate to access to specialised facilities.
2. In the methods it would be helpful if the authors could identify the cage type they used. By small gaps do they mean the spaces under the feed and water hoppers?
3. The authors note that no environmental enrichment can be used that might fully obscure the animal. Can the authors confirm that some nesting material was available but presumably was limited in quantity?
4. The authors used cages in light tight boxes. Have they tested their PIR system in more conventional animal caging?

I have read this submission. I believe that I have an appropriate level of expertise to confirm that it is of an acceptable scientific standard.

# Response

**Published:** 2017-04-11

**Authors:** Laurence Brown

**Affiliations:** University of Oxford, UK

Dear Professor Wells

 Thank you for your review. We hope the revised manuscript will address the important points you raised:

1. We agree this should be clarified.  We have rewritten this paragraph in the revised manuscript.
2. This is correct. In some cages we have food and water hoppers mounted externally, so no further modification was needed, but where the water bottles and food are integrated into the wire of the cage lid, a Perspex block was used to ensure the mice were active and nested in view of the sensor.  We have added the cages types used in the paper to the revised version.
3. We provided nesting materials for all of the mice in the study and in preliminary work. This was paper-based Sizzle-Nest, or cotton fibre Nestlets, with a minimum of 10g in each cage (in excess of that required for thermoregulation by mice with similar materials, Gaskill et al 2012, PLOS ONE, ( http://dx.doi.org/10.1371/journal.pone.0032799).

1. We are currently collecting data with collaborators in a range of cage sizes and types of racking. For some types of cage, particularly those with food and water hoppers recessed in the centre of the cage lid rather than at one end, it will be difficult to ensure complete coverage of the cage. Individually-ventilated cages (IVCs) would need modification for the integration of PIR sensors, as the polycarbonate material most are constructed with will block the 5-10µm infrared wavelengths of body heat.
